# The Architectural Chromatin Factor High Mobility Group A1 Enhances DNA Ligase IV Activity Influencing DNA Repair

**DOI:** 10.1371/journal.pone.0164258

**Published:** 2016-10-10

**Authors:** Ilenia Pellarin, Laura Arnoldo, Silvia Costantini, Silvia Pegoraro, Gloria Ros, Carlotta Penzo, Gianluca Triolo, Francesca Demarchi, Riccardo Sgarra, Alessandro Vindigni, Guidalberto Manfioletti

**Affiliations:** 1 Department of Life Sciences, University of Trieste, Trieste, Italy; 2 International Centre for Genetic Engineering and Biotechnology (ICGEB), Trieste, Italy; 3 Laboratorio Nazionale Consorzio Interuniversitario Biotecnologie (LNCIB), AREA Science Park, Trieste, Italy; 4 Edward A. Doisy Department of Biochemistry and Molecular Biology, Saint Louis University School of Medicine, Saint Louis, Missouri, United States of America; University of South Alabama Mitchell Cancer Institute, UNITED STATES

## Abstract

The HMGA1 architectural transcription factor is an oncogene overexpressed in the vast majority of human cancers. HMGA1 is a highly connected node in the nuclear molecular network and the key aspect of HMGA1 involvement in cancer development is that HMGA1 simultaneously confers cells multiple oncogenic hits, ranging from global chromatin structural and gene expression modifications up to the direct functional alterations of key cellular proteins. Interestingly, HMGA1 also modulates DNA damage repair pathways. In this work, we provide evidences linking HMGA1 with Non-Homologous End Joining DNA repair. We show that HMGA1 is in complex with and is a substrate for DNA-PK. HMGA1 enhances Ligase IV activity and it counteracts the repressive histone H1 activity towards DNA ends ligation. Moreover, breast cancer cells overexpressing HMGA1 show a faster recovery upon induction of DNA double-strand breaks, which is associated with a higher survival. These data suggest that resistance to DNA-damaging agents in cancer cells could be partially attributed to HMGA1 overexpression thus highlighting the relevance of considering HMGA1 expression levels in the selection of valuable and effective pharmacological regimens.

## Introduction

The unrestricted accumulation of DNA damages is one of the major driving causes leading to genomic instability and, as a direct consequence, to neoplastic transformation and cancer development. To counteract this, cells have evolved multiple checkpoints and pathways in order to rapidly sense DNA damages and provide efficient repair mechanisms [1]. However, there are several pathological conditions in which key repair system components are impaired leading to accumulation of DNA damages and cancer predisposition. Examples are the Bloom and Werner syndromes whose patients are defective in the *BLM* and *WRN* helicase genes, respectively [2].

The High Mobility Group A proteins HMGA1 (with the two splicing variants HMGA1a and HMGA1b) and the highly related HMGA2, are a family of chromatin factors playing essential physiological functions during embryonic development and, in a restricted manner, also in some differentiated adult tissues. Indeed, HMGA are highly expressed during embryogenesis [3] and the interference with their expression during this process has profound phenotypic effects [4, 5]. The expression levels of HMGA proteins in differentiated cells are very low if not almost undetectable. Nonetheless, they perform essential functions, one of the most striking examples being their role in the expression of the insulin receptor [6]. In addition to their physiological role, HMGA are highly expressed when cells undergo neoplastic transformation and there are several experimental evidences that clearly support a causal role of HMGA in cancer development [7]. HMGA proteins are architectural chromatin transcription factors that, through their ability of interacting with both proteins and DNA, can modulate chromatin structure and organize stereospecific macromolecular complexes having a profound impact on gene expression [8]. Indeed, many of the HMGA oncogenic activities have been explained through gene transcription regulatory mechanisms. For example, HMGA proteins bind p53 impairing its transcriptional activity [9], enhance cell cycle progression modulating the transcription of cyclin A [10], increase cell motility regulating the transcription of cyclin E2 [11, 12], and interact with pRB displacing HDAC1 from the pRB/E2F1 complex hence promoting E2F1 activation [13].

Among their various oncogenic activities, HMGA proteins play a role in DNA repair. HMGA1 protein expression level correlates with the onset of chromosomal rearrangements. Initially, this effect was associated to the peculiar organizing activity of HMGA1 towards chromatin structure [14]. However, HMGA1 has recently been shown to interfere with the Nucleotide Excision Repair (NER) pathway. This effect has been partially explained with a direct interference of DNA-bound HMGA1 with the recruiting of NER repair factors [15, 16] and with a direct downregulation of the expression of the Xeroderma Pigmentosum Group A protein, one of the NER DNA damage repair system key factors [17]. In addition, HMGA2 interferes with the NER pathway, downregulating the expression of the excision repair cross-complementing rodent repair deficiency, complementation group 1 protein by binding to the regulatory sequences of this gene [18]. Moreover, HMGA1 was shown to be involved in the downregulation of the Breast Cancer Gene 1 (BRCA1) adding a further negative hit to the functionality of the NER pathway [19]. In agreement with its proposed role in DNA repair, HMGA1 overexpression leads to a sensitization to DNA damaging drugs, such as cisplatin or bleomycin [20].

Recent studies point to an additional role of HMGA in double-strand break (DSB) repair. DSBs are among the most dangerous forms of DNA lesions because they can lead to gross DNA chromosomal rearrangements with drastic effects on cell phenotype [21]. For this reason, distinct DNA repair pathways have been evolved to cope with DSBs, including the error-free homologous recombination (HR) and the error-prone non-homologous end joining (NHEJ) pathways [21]. HMGA proteins have been linked with two of the key factors involved in DSBs surveillance and repair, ATM (Ataxia Telangiectasia Mutated) and DNA-PK (DNA-dependent Protein Kinase). These two factors belong to the Phosphatidylinositol 3-kinase-related kinases (PIKKs) family and orchestrate, through their activity, the onset of DSB damage responses [22]. HMGA1 was found to be in complex with ATM *in vivo* and to be specifically phosphorylated by this kinase [23]. Moreover, HMGA1 overexpressing cells display a decreased survival following IR treatment, suggesting a negative modulatory role for HMGA1 in DSB repair [23]. In agreement with these data, HMGA2 overexpressing cells display an increase in basal level of γ‒H2AX and they are more sensitive to DSBs damage, such as X-ray and cisplatin [24]. A clear connection of HMGA2 with the NHEJ comes from the observation that the presence of HMGA2 alters the proper phosphorylation-mediated activation of DNA-PK, leading to a sustained persistence of DNA-PK at DSB sites [25]. These events have been linked to an increase of basal phosphorylation of H2AX and a delayed clearance of the same from DSB sites. Moreover, HMGA2 overexpression was shown to cause an increased genomic instability with a more pronounced accumulation of spontaneous chromosome aberrations [25]. However, a molecular mechanism involving HMGA proteins in DSBs repair is still missing.

In this work we show that HMGA1 is associated with the macromolecular complex involved in NHEJ that is formed by the key holoenzyme DNA-PK (Ku70/Ku80/DNA-PKcs) and several associated co-factors, among which Ligase IV and XRCC4. We demonstrate that HMGA1a and HMGA2 proteins are *in vitro* substrates for DNA-PK and identify their phosphorylation sites. Importantly, we show that HMGA1a enhances the activity of Ligase IV and that high levels of expression of HMGA1 protects breast cancer cells from a DSB genotoxic insult favouring the clearance of DSBs and cell survival.

## Results

### HMGA1a forms a complex with the NHEJ machinery and is a substrate for DNA-PK

**Fig 1:**
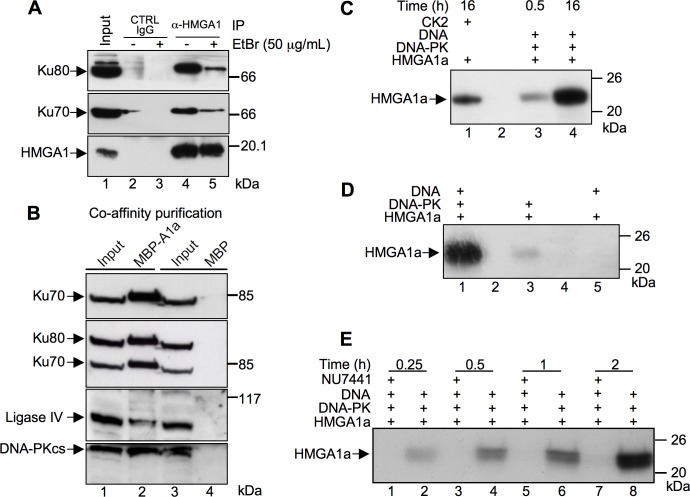
HMGA1a associate with the NHEJ DNA repair protein machinery and is a DNA-PK substrate. A. Co-immunoprecipitation (Co-IP) assay of endogenous HMGA1, Ku70, and Ku80 proteins on MDA-MB-231 cells in the presence of absence of Ethidium Bromide (EtBr). Cell lysates were immunoprecipitated with α-HMGA1. The cell lysates (input) and immunoprecipitates were analyzed by immunoblotting with antibodies as indicated. B. HMGA1a fused to MBP (MBP-A1a) or the MBP alone were produced by transient transfection in HEK 293T cells. Cellular lysates (input, lanes 1 and 3) were incubated with amylose resin and affinity captured MBP-HMGA1a and MBP proteins recovered. Bound proteins were eluted by SDS sample buffer, separated by SDS-PAGE (T = 10%), and analyzed by western blot using antibodies specific for Ku70, Ku80 (after Ku70 recognition), Ligase IV, and the catalytic subunit of DNA-PK, DNA-PKcs. C. Recombinant HMGA1a protein was subjected to a phosphorylation assay in presence of [γ-^32^P] ATP with DNA-PK for 0.5 and 16 h (lanes 3 and 4, respectively) and CK2 for 16 h (lane 1). D. Recombinant HMGA1a was subjected to a phosphorylation assay for 16 h with a complete DNA-PK reaction mix (lane 1), without activating DNA (lane 3), or without DNA-PK itself (lane 5). E. Time course phosphorylation assay (0.25, 0.5, 1, and 2 hours) performed with recombinant HMGA1a in the presence (lanes 1, 3, 5, and 7) or absence (lanes 2, 4, 6, and 8) of a specific DNA-PK inhibitor (NU7441–50 nM). Phosphorylated proteins were separated by SDS-PAGE (T = 15%) and ^32^P incorporation visualized by autoradiography. Protein molecular markers (kDa) are indicated on the right.

*In vitro* proteomic screenings, previously performed in our laboratory, brought to light that HMGA architectural transcription factors are direct molecular partners of Ku80 and Ku70 factors [26, 27]. In order to deepen these findings we performed co–immunoprecipitations to assess whether HMGA1 interacts with these proteins *in vivo* as well. Etidium Bromide (EtBr) was included in order to evaluate whether the interactions were direct or DNA–dependent. Fig 1A shows that HMGA1 is able to associate with Ku70 and Ku80 (lane 4) and that the complex is still present, albeit at lower levels, when EtBr is included in the lysates (lane 5) suggesting that the association is dependent on protein-protein interaction although the presence of DNA could increase the amount of the complex formed. We then asked whether HMGA1 could associate also with other two members of the NHEJ machinery, i.e. Ligase IV and DNA-PKcs. Vectors expressing HMGA1a in fusion with MBP (Maltose Binding Protein, MBP-A1a) or MBP alone, as a negative control, were transfected in HEK 293T cells and soluble protein complexes were purified by affinity chromatography using amylose resin (Co-AP). HMGA1a bound proteins were SDS-PAGE and western blot analyzed using antibodies specific for Ligase IV and DNA-PKcs together with Ku70 and Ku80 that were included as controls. Fig 1B shows that all the analyzed proteins were efficiently co-purified together with MBP-HMGA1a (lane 2) but not with MBP alone (lane 4).

DNA-PK phosphorylates a plethora of substrates, most of which are involved in the DNA damage response. Thus, in order to provide additional evidence that HMGA1 could associate with the NHEJ complex, we tested whether HMGA1a could be a DNA-PK substrate. There are three DNA-PK canonical SQ consensus sites within HMGA1a sequence (S8Q, S43Q, and S98Q). We performed *in vitro* phosphorylation assays using recombinant HMGA1a, commercially available DNA-PK purified from HeLa cells, and radiolabeled [γ-^32^P] ATP (Fig 1C). The autoradiography of phosphorylated HMGA1a protein separated by SDS-PAGE clearly showed the incorporation of ^32^P in HMGA1a after 0.5 or 16 hours of phosphorylation (lanes 3 and 4, respectively). Phosphorylation performed with casein kinase two (CK2, lane 1), which is a well-known kinase that phosphorylates HMGA proteins, was used as a positive control [28, 29]. Since DNA-PK activity depends on the presence of DNA, as a control we evaluated whether DNA-PK was able to phosphorylate HMGA1a in its absence. Our results showed that this is not the case as the omission of DNA caused a strong decrease of phosphate incorporation (Fig 1D, compare lane 3 with lane 1). Moreover, a phosphorylation reaction performed in the presence of DNA but in the absence of DNA-PK showed that HMGA1a modifying kinases are not co-purified with the DNA used to activate DNA-PK (Fig 1D, lane 5). As a further control of DNA-PK activity towards HMGA1a we performed a time course phosphorylation experiment using NU7441, a potent DNA-PK inhibitor [30]. The inhibitor specifically abrogated HMGA1a phosphorylation by DNA-PK (Fig 1E, compare lanes 1, 3, 5, and 7 with lanes 2, 4, 6, and 8, respectively). Collectively, these experiments clearly show that HMGA1a is a bona fide DNA-PK substrate.

**Fig 2:**
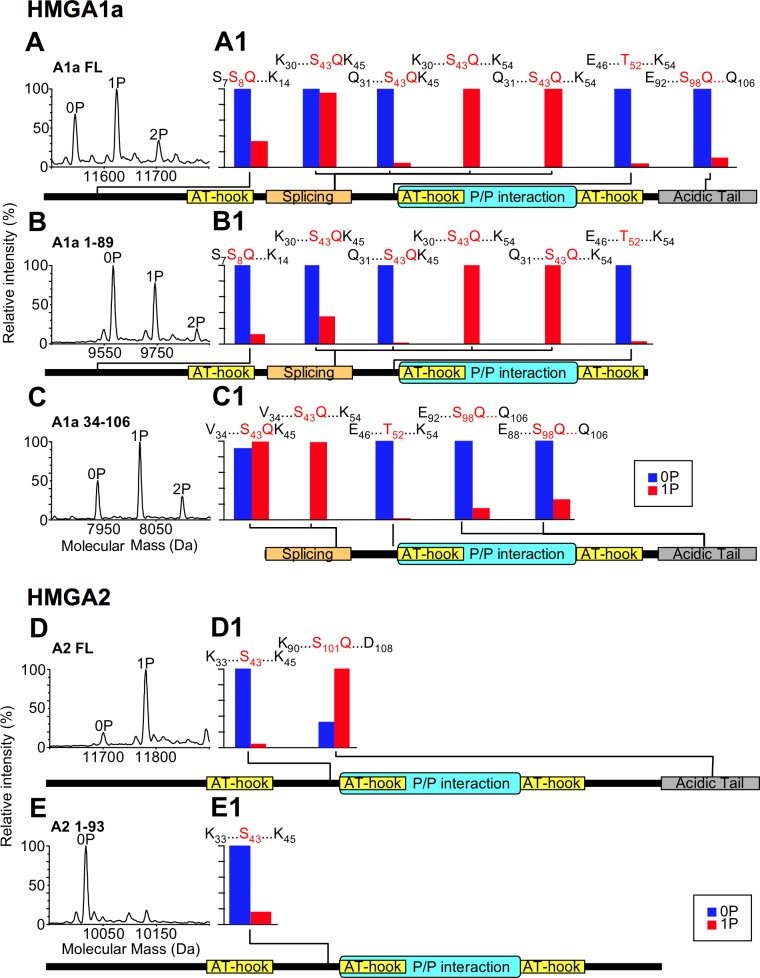
HMGA1a and HMGA2 are phosphorylated by DNA-PK. Different recombinant HMGA1a protein forms (full-length (FL), 1–89, and 34–106) and HMGA2 protein forms (full-length (FL) and 1–93) were phosphorylated by DNA-PK for 16 h and analyzed by LC-MS. Reconstructed mass spectra of the phosphorylated proteins are reported in panels A-E; P indicates the phosphate group. Each protein form was digested by trypsin and peptides analyzed by LC-MS/MS. For each HMGA form (panels A1-E1), a schematic view reports the mass/charge (m/z) relative intensity of phosphorylated peptides (red bars) in comparison with their unmodified counterparts (blue bars). The identities of these peptides (given by first and last aminoacid residue), together with their modified S/T residues, are indicated. A schematic representation of the various HMGA1a forms allows to map the phosphorylation sites with respect to HMGA functional domains (AT-hook: DNA-binding domain; Splicing region: the aminoacid region lacking in HMGA1b splicing isoform; P/P interaction: protein/protein interaction domain; Acidic tail: acidic C-terminal tail).

Next, we performed LC-MS and LC-MS/MS analyses to obtain more precise information on the modified sites. HMGA2 was also previously shown to play a role in modulating the DNA-PK mediated response following DNA damage [25]; thus we sought to test whether HMGA2 is a DNA-PK substrate as well. HMGA1a has three DNA-PK canonical consensus sites (S8Q, S43Q, and S98Q) and HMGA2 has only one, located on its C-terminal tail (S101Q). We performed phosphorylation assays using full-length and truncated HMGA1a and HMGA2 forms, in which single consensus sites for DNA-PK are lacking (Fig 2). In the reconstructed mass spectra (Molecular Mass vs. Relative Intensity (%)) of Fig 2, panels A–C showed that full-length HMGA1a was mainly mono-phosphorylated (1P) and also the other two truncated forms of HMGA1a appear to be in the same phosphorylation status. A minor fraction of HMGA1a forms was also bi-phosphorylated (2P). On the other hand, reconstructed mass spectra of HMGA2 forms (Fig 2D and 2E) showed that full-length HMGA2 was completely mono-phosphorylated and its C-terminal truncated form was unmodified. Given that the phosphorylation status of all HMGA1a forms is the same, we hypothesized that the major phosphorylation site was within the 34–89 region. The presence of bi-phosphorylated forms suggests that HMGA1a can be phosphorylated at multiple sites, although with a lower efficiency. Data regarding HMGA2 unambiguously showed that the phosphorylation site was located on the acidic C-terminal tail. These conclusions are enforced by LC-MS/MS analyses performed on tryptic peptides obtained from full-length (FL) and truncated HMGA forms (Fig 2A1–2C1 (HMGA1a) and Fig 2D1 and 2E1 (HMGA2), and S1 MS Data File). These analyses, which exploit the phosphorylation site assignment by the Mascot software, strongly suggest that the major phosphorylation sites on HMGA1a is S43Q and that HMGA2 is phosphorylated at the level of S101Q. In order to provide additional evidences that the phosphorylation sites are those indicated by Mascot, we compared mass/charge (m/z) relative intensities of each phosphorylated peptide detected in our analyses (red bars) with those of unmodified counterparts (blue bars) (Fig 2, panels A1–C1 (HMGA1a) and panels D1 and E1 (HMGA2), and S1 MS Data File). In the case of HMGA1a, only those phosphorylated peptides containing the phosphorylated S43Q consensus site have higher m/z intensities with respect to their unphosphorylated forms. As concern HMGA2, the C–terminal peptide phosphorylated at S101Q is the only peptide with a higher intensity of the phosphorylated form.

### HMGA1a enhances the activity of Ligase IV

**Fig 3:**
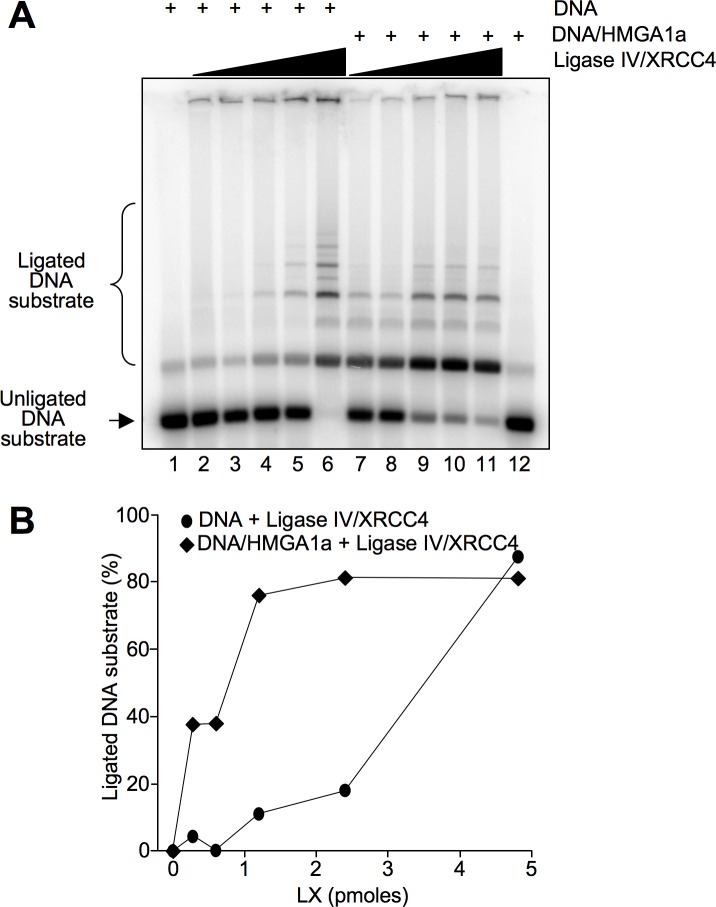
HMGA1a enhances Ligase IV activity. A. DNA ligation was assayed using increasing quantities of DNA Ligase IV/XRCC4 (LX) complex (0, 0.3, 0.6, 1.2, 2.4, and 4.8 pmoles) either incubated with DNA alone (lanes 2–6) or with DNA pre-incubated with HMGA1a (1.2 pmoles, lanes 7–11). The DNA substrate (a double-stranded DNA fragment of 442 bp with 4 bp overhangs) and the ligated DNA multimers of different length were separated in an agarose gel. Lanes 1 and 12 show DNA or DNA/HMGA1a alone as controls, respectively. The figure shows a representative ligation assay. B. Quantification of ligation assay shown in A. The percentage of ligated DNA substrate is plotted as a function of the quantity of DNA Ligase IV/XRCC4 complexes (pmoles).

Results reported above show that HMGA1a could be associated with the NHEJ complex to which participates also Ligase IV. Since the final step of NHEJ is ligation of the DNA broken ends by Ligase IV assisted by XRCC4 (usually referred to as LX complex) we tested whether HMGA1a could influence LX activity. *In vitro* end-joining (ligase) assays demonstrated that when the DNA is pre-incubated with HMGA1a and then LX is added, HMGA1a stimulates the ligation activity of Ligase IV (Fig 3) especially at lower LX concentrations (compare lanes 2, 3, and 4 with lanes 7, 8, and 9).

**Fig 4:**
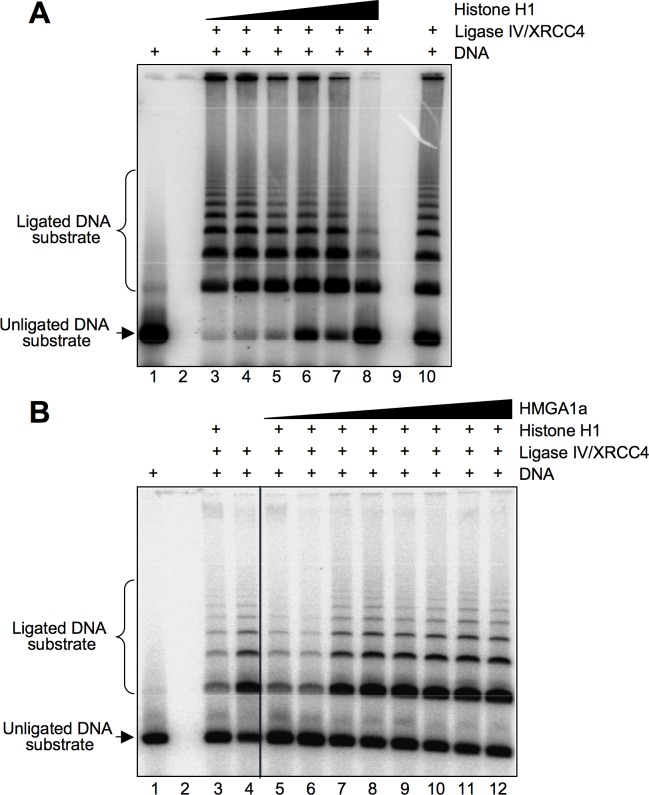
HMGA1a counteracts the repressive role of histone H1 with respect to Ligase IV/XRCC4 activity. A. DNA ligation was assayed using fixed amount of Ligase IV/XRCC4 (1.2 pmoles) and increasing quantities of histone H1 (0.1, 0.2, 0.4, 0.8, 1.2, and 2.4 pmoles, lanes 3–8). The unligated DNA substrate is shown in lane 1 and the activity of Ligase IV/XRCC4 alone is shown in lane 10. B. DNA ligation was assayed using fixed amount of Ligase IV/XRCC4 and histone H1 (1.2 and 2.4 pmoles, respectively) and increasing amounts of HMGA1a (0.2, 0.4, 0.6, 0.8, 1.0, 1.2, 2.4, and 4.8 pmoles, lanes 5–12). The unligated DNA substrate is shown in lane 1, the Ligase IV/XRCC4 activity in the presence of histone H1 (2.4 pmoles) is shown in lane 3, and the Ligase IV/XRCC4 alone is shown in lane 10. The figure shows representative ligation assays.

HMGA1 is known to compete with histone H1 for DNA binding [31] and histone H1 has a dual activity with respect to DNA ligation efficiency. At low concentrations it enhances Ligase IV activity by aiding in the bridging of DNA ends [32]. Conversely, at higher concentrations histone H1 acts as an occluding factor promoting the formation of aggregates and inhibits Ligase IV activity [33]. Thus, we tested whether HMGA1a/histone H1 DNA binding competition could relieve the histone H1 occluding effect. We reproduced the experimental conditions adopted to detect an inhibitory role of histone H1 versus Ligase IV activity [32, 33] and tested the effect of HMGA1a under these conditions. Results showed that HMGA1a was able to relieve the inhibitory effect of histone H1 towards Ligase IV activity (Fig 4).

### HMGA1 enhances the recovery from DSB and the survival capacity of DSB-damaged cells

**Fig 5:**
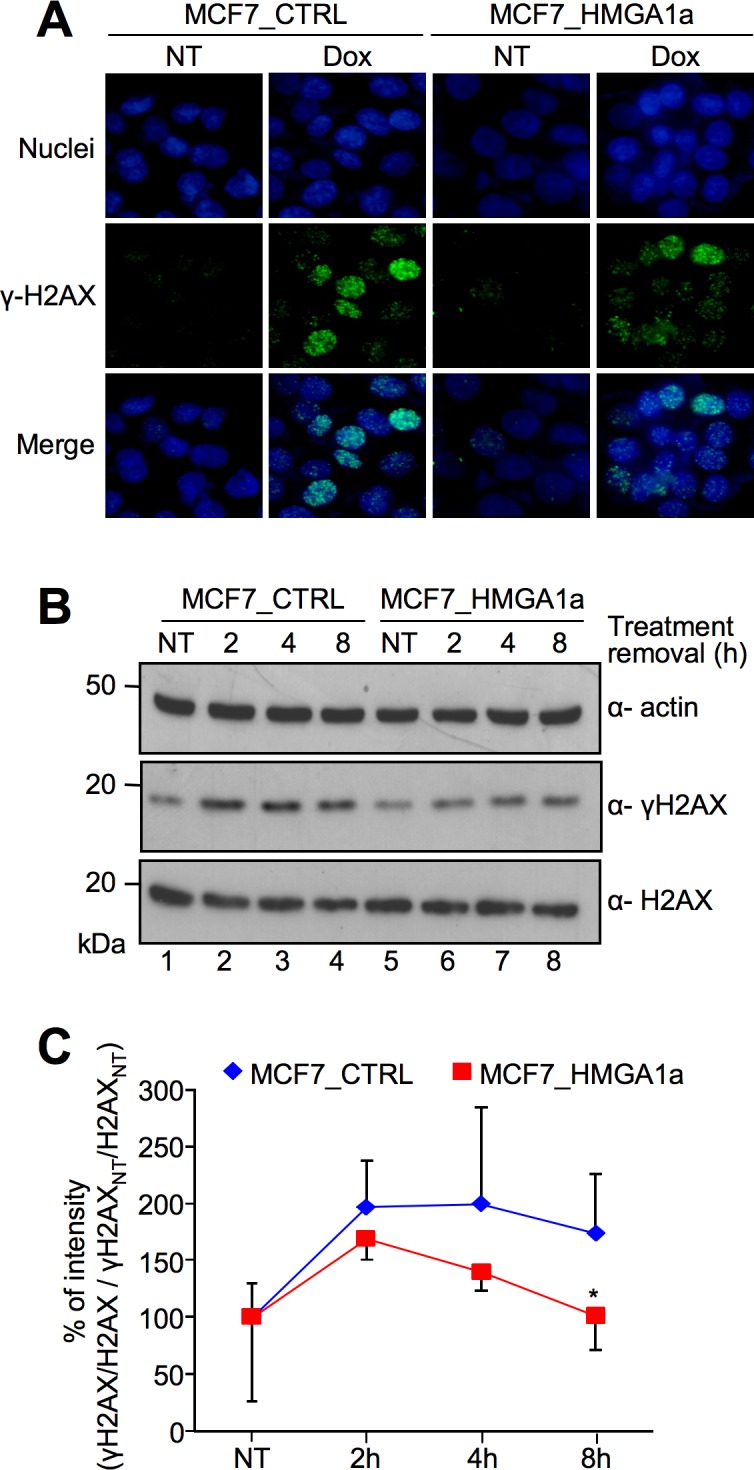
HMGA1a overexpression alters the kinetic of DNA repair in MCF-7 breast cancer cells. A. Immunofluorescence analyses for the visualization of γ-H2AX foci formation in MCF7_CTRL and MCF7_HMGA1a cells exposed to 1 μM doxorubicin (Dox) at 2 hours after treatment removal. Not treated cells (NT) are shown as a control. Nuclei are stained with Hoechst (blue). Images were acquired using a Nikon Eclipse E800 epifluorescence microscope with a 40X objective coupled with a Nikon DXM1200 camera. The mean number of foci/nuclei after doxorubicin tretment is 20.0 and 20.6 for MCF7_CTRL and MCF7_HMGA1a, respectively. Nuclei were manually counted while foci were counted using ImageJ after applying the same threshold for all images, then using the Analyze Particles tool. B. Western blot analyses showing γ-H2AX and H2AX expression levels in MCF7_CTRL and MCF7_HMGA1a cells. Cells were treated with 1 μM doxorubicin for 2 hours and lysates were collected at different time points after treatment removal (not treated—NT and 2, 4, and 8 hours after treatment). Actin was used as an internal normalization. Protein molecular markers (kDa) are shown on the left. C. Graph showing the quantitative evaluation of γ-H2AX induction following the doxorubicin treatment described in B. The reported points are the mean percentage value of four independent experiments ± SD. For each time point, γ-H2AX percentage (%) of intensity (based on densitometric analysis of western blot data) is calculated with the following criteria: (γ-H2AX/H2AX / γ-H2AX_NT_/H2AX_NT_) x 100, P value: * < 0.05.

To test *in vivo* the effect of HMGA1 on the DSB repair, we used the low HMGA1-expressing breast cancer MCF7 cells stably transfected with a plasmid expressing a HA-tagged HMGA1a (MCF7_HMGA1a) and control cells transfected with the empty vector (MCF7_CTRL) (S1 Fig, panel A). FACS analyses showed that wild type MCF7, MCF7_CTRL, and MCF7_HMGA1a cells were mainly present in the G0/G1 phase of the cell cycle (>70%) (S1 Fig, panel B), thus indicating that when treated with DSB inducing agents, these cells would repair DNA damage mainly by NHEJ and not by HR. The DNA damage response (DDR) kinetics is usually visualized by the appearance of γ-H2AX foci and measured by H2AX phosphorylation levels. We treated MCF7_HMGA1a and MCF7_CTRL with doxorubicin, ascertained the appearance of γ-H2AX foci (Fig 5A) and semi–quantitatively followed the recovery of γ-H2AX in a time course experiment by western blot analyses (Fig 5B and 5C). The results clearly indicate that MCF7_CTRL cells had a prolonged presence of γ-H2AX while in MCF7_HMGA1a cells H2AX phosphorylation was promptly recovered, being the H2AX phosphorylation level of MCF7_HMGA1a cells at 8 hours significantly lower than that of the MCF7_CTRL cells.

**Fig 6:**
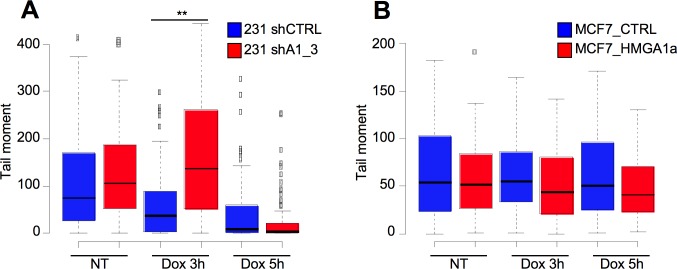
HMGA1 expression lowers the presence of DNA double-strands breaks. A. Quantitative evaluation of neutral comet assays performed on MDA-MB-231_shCTRL and MDA-MB-231_shA1_3 (A) and MCF7_CTRL and MCF7_HMGA1a cells (B) treated with doxorubicin (Dox) for 2 hours and left recover DNA damage for 3 and 5 hours. Not treated cells (NT). Box plot showed the tail moment. P value: ** < 0.01.

To see whether the HMGA1–linked rapid disappearance of γ-H2AX corresponds to a lower presence of DSBs, we compared the DNA integrity of MCF7_CTRL cells and MCF7_HMGA1a cells after DNA damage induction by neutral comet assay that allow detection of DSBs. In addition, to have more robust data, we used also a highly expressing HMGA1 breast cancer cell line, MDA-MB-231, that has been silenced for HMGA1 expression through shRNA (MDA-MB-231_shA1_3; [11] and S1 Fig panel C). Fig 6A shows that at 3 hours after doxorubicin removal, MDA-MB-231 cells silenced for HMGA1 expression (MDA-MB-231_shA1_3) recover significantly slower than control cells (MDA-MB-231_shCTRL), as assessed by the higher mean tail moment measured with the comet assay. Fig 6B shows the mirror experiment performed with MCF7_CTRL and MCF7_HMGA1a cells. HMGA1a overexpressing cells show, at both time points after doxorubicin removal, a lower—albeit not statistically significant—tail moment with respect to control cells. Altogether these data suggest an increased DSB repair capability in HMGA1 expressing cells.

**Fig 7:**
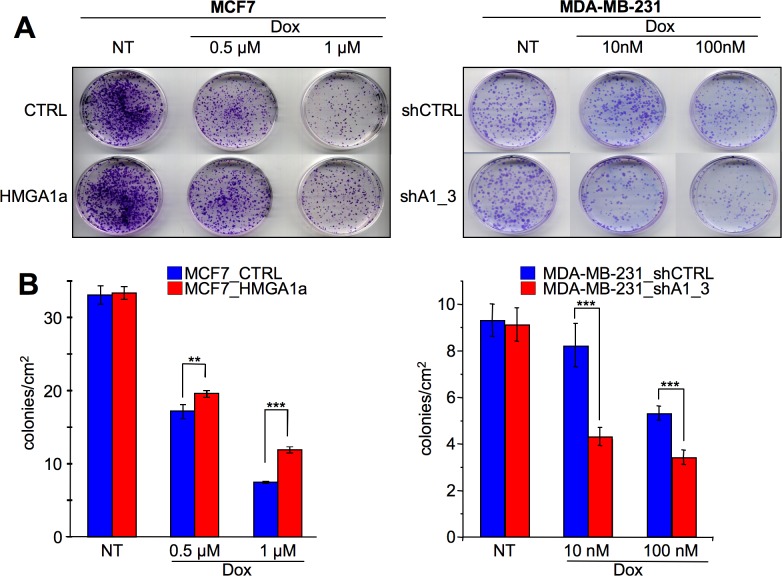
HMGA1 confers breast cancer cells a survival advantage with respect to DNA damaging agents. A. Colony formation assay performed on MDA-MB-231_shCTRL and MDA–MB–231_shA1_3 cells (A) and MCF7_CTRL and MCF7_HMGA1a cells (B). Cells were treated with doxorubicin (Dox) to induce DNA damage (not treated—NT), then left to grow, fixed, stained with 0.5% crystal violet, and counted. In the upper panel is shown the representative images of the colony assay, in the lower panel is shown the quantification of the colony formation assay as the mean number of colonies/cm^2^ ± SD (n = 4). P value: ** < 0.01; *** < 0.001.

The presence of unrepaired DNA is incompatible with cell survival. To evaluate whether HMGA1 expression could provide cells with a survival advantage towards DNA damaging agents, we treated both MDA-MB-231_shCTRL and MDA-MB-231_shA1_3 cells (HMGA1 silencing condition) (Fig 7A) and MCF7_CTRL and MCF7_HMGA1a cells (HMGA1a overexpression condition) (Fig 7B) with doxorubicin and evaluated their survival rate by colony formation assay. In agreement with the comet assay, HMGA1 expression is significantly linked to a higher rate of cell survival (Fig 7A and 7B).

## Discussion

DNA damage routinely occurs within the chromatin context and various repair mechanisms have to deal with the fact that DNA is not freely accessible. Most of the DNA is tightly wrapped around core histone octamers forming nucleosomes that are further compacted by histone H1-mediated higher order structures. Among the various proteins participating in chromatin organization, HMGA (High Mobility Group A) proteins play a pivotal role in conferring plasticity to chromatin affecting DNA structure dynamics [34, 35].

HMGA proteins have a plethora of interacting partners and, adopting different protein/protein interaction discovery approaches, we were able to evidence the interaction of HMGA with the DNA binding subunits of DNA-PK, i.e. Ku70 and Ku80 [26, 27]. In this work, we provide evidence that HMGA1 could associate *in vivo* with the NHEJ complex. DNA-PK assembles onto the DNA ends of double-strand breaks, phosphorylates several factors involved in the DNA repair process, but the functional consequences of all these phosphorylation events are not fully understood [36]. As an additional proof that HMGA1a associates with the NHEJ complex, we were able to show that HMGA proteins (both HMGA1a and HMGA2) are *in vitro* DNA-PK substrates. Our data are in line with previous work showing that HMGA1 and HMGA2 interact with and are substrates for the other two PIKKs involved in DNA repair, i.e. ATM (HMGA1b: interaction and substrate) and ATR (HMGA2: interaction) [23, 37]. Further evidence supporting the idea that HMGA have a functional relationship with the NHEJ machinery comes from mass spectrometry analysis of the commercially available DNA-PK that we used in our experiments. The DNA–PK that we used in our *in vitro* phosphorylation assays is purified from HeLa cells by means of several chromatographic steps performed in native conditions to preserve the enzymatic activity. We analyzed DNA-PK by means of mass spectrometry performing a tryptic digestion and LC–MS/MS analysis (data reported in S1 MS Data File). In addition of identifying DNA–PKcs, we identified one of its co–factors (i.e. XRCC6 –Ku70) and, very interestingly, we detected also several members associated with DNA–PK, both as substrates and interactors, i.e. histone H1, heterogeneous nuclear ribonucleoprotein A1, non-POU domain-containing octamer-binding protein, splicing factor, proline- and glutamine-rich and, notably, HMGA2 itself (references reported in S1 MS Data File).

What could be the function of HMGA1 within the DNA-PK complex? We focused on Ligase IV and investigated whether HMGA1a could influence Ligase IV activity. Our *in vitro* ligation assays suggested that HMGA1a, when pre-bound to DNA, could enhance Ligase IV activity. We speculate that the multiple DNA binding domains of HMGA1a could tether together AT-rich DNA extremities thus favouring DNA joining. We are aware that this Ligase IV enhancing activity is similar to that described for HMGB proteins [38–41], with the peculiar difference that being HMGA proteins expression specifically associated with embryonic development and neoplastic transformation their contribution could be specific and relevant in these phases. It is worthwhile to evidence that our *in vitro* phosphorylation assays performed with purified DNA-PK suggest that the HMGA1a protein could enhance the autophosphorylation activity of DNA-PK (S2 Fig).

The idea that HMGA1a is involved in modulating the Ligase IV end joining efficiency is further corroborated by experiments performed in the presence of histone H1 at concentrations inhibiting Ligase IV activity. HMGA1 is a well-known and very efficient histone H1 DNA-binding competitor [31] and we showed that HMGA1a efficiently counteracts the repressive role of H1 with respect to Ligase IV.

Based on these *in vitro* observations, we hypothesized that the expression of HMGA1a should provide cells an enhanced DNA DSBs recovery pathway. To this end, we treated two breast cancer cell models, one that overexpress HMGA1a and the other where the expression of HMGA1 has been silenced, with doxorubicin, a DSB inducer and one of the most effective agents for the treatment of breast cancer patients whose efficacy, however, is often compromised by resistance development [42, 43]. Accordingly with *in vitro* data, HMGA1 expressing cells have a faster DNA damage recovery kinetics, a less extent of DSBs, and more importantly, their survival rate is enhanced with respect to control cells.

As remarked in the introduction, there are several studies demonstrating a role for HMGA proteins in the modulation of DNA damage response systems [15–20, 23–25, 37, 44–47] and in particular as concern DSB repair [23–25, 44, 45]. However, the HMGA role in these pathways is still unclear. A sensitization linked to HMGA1 expression cells was evidenced in mouse ES (HMGA1 KO vs. HMGA1 WT) and in MCF7 (HMGA1b forced overexpression) cells following cisplatin or bleomycin treatments [20, 23] while a HMGA1 protective role with respect to ionizing radiation (IR) was later shown on mouse ES (HMGA1/2 KO vs. HMGA1/2 WT) and on cancer thyroid cells (FRO and FRO silenced for HMGA1) [44]. The HMGA2 protein was shown to increase the level of basal H2AX phosphorylation thus increasing the threshold for DNA repair activation and sensitizing cells to DNA damaging agents [24] and later to have an impairment role with respect to DNA-PK activity [25].

The DSB impairment role for HMGA1 fits well with the oncogenic role of HMGA proteins that, by impairing the proper recovery from DNA damage, could promote the progressive accumulation of DNA lesions, which eventually could provide selective advantage to cancer cells. This activity however is not in accordance with the role of HMGA1 during embryonic development, a phase that is characterized by a high cell proliferation rate that has to be associated with a strictly controlled maintenance of genomic integrity. In addition, also cancer cells must provide themselves with mechanisms enabling them to protect from an over-accumulation of DNA lesions, which if not properly controlled, would ultimately lead cancer cells to death.

HMGA proteins have been widely linked with stemness maintenance and are recognized as important players in modulating cancer stem cells (CSCs) properties [11, 48–52]. As a matter of fact, also CSCs display an unusual resistance to DNA targeting radio- and chemo–therapy [53, 54], fact that is due to several mechanisms, which ultimately converge in an enhanced DNA-repair capability [53, 54].

A possible explanation for the contradictory findings reported in literature could reside in the molecular context. The HMGA proteins mentioned above have a plethora of molecular partners that could influence their activity, thus shifting HMGA1 function from a pro-DNA damaging to a DNA-protective role. Another possible explanation, as suggested by Bullerdiek and Rommel [55], could reside in different HMGA expression levels: depending on their amount they could exert opposite effects as previously observed for histone H1 [32, 33]. Another intriguing aspect is that HMGA proteins are not fully equivalent [56, 57]. We hypothesize that the functional outcome of DNA-PK phosphorylation would be very different between HMGA1 and HMGA2. Indeed, HMGA1a is mainly phosphorylated by DNA-PK at the level of S43Q, a phosphorylation site that is located between the first and second AT-hook DNA-binding domain that has a profound impact on the DNA binding ability of HMGA1a [58]. On the contrary, HMGA2 is phosphorylated at the level of the C-terminal tail (S101Q), and this phosphorylation has not the same role as that described for HMGA1a [28].

In summary, we provide additional evidences for an involvement of HMGA proteins in DNA repair processes and highlight the importance of taking into consideration and further explore HMGA role in the modulation of DNA damage repair pathways, especially considering their functional outcome in relationship with pharmacological treatment targeting DNA integrity. Moreover, we suggest the possibility that HMGA proteins could have different impacts with respect to DNA repair.

## Materials and Methods

### Co–affinity purification and co–immunoprecipitation

Co–affinity purifications and co–immunoprecipitations were performed as previously described [31, 59] with the following modifications only for co–IP analysis: 500 μg lysate (untreated or incubated with 50 μg/ml Etidium Bromide), 4 μg antibody (both α–HMGA1 and aspecific IgG). Proteins were separated by 10% or 15% SDS-PAGE and transferred onto nitrocellulose membrane for immunoblotting. Ku70, Ku80, Ligase IV, and DNA PKcs were recognized using specific antibodies (Santa Cruz Biotechnology, Santa Cruz, CA, USA). The α–HMGA1 antibody is a rabbit polyclonal antibody developed in our laboratory.

### Recombinant protein production and purification

Recombinant HMGA proteins were produced in *E*. *coli*, extracted, HPLC-purified, and quantified as previously described [60].

### In vitro phosphorylation assays

*In vitro* phosphorylation of HMGA1a protein by CK2 was performed by incubating 5 μg of recombinant protein with 100–250 Units of CK2 (New England BioLabs) in 50 μL of reaction volume (20 mM Tris/HCl pH 7.5, 50 mM KCl, 10 mM MgCl_2_, and 3.3 μM [γ-^32^P] ATP) at 37°C for 0.5 or 16 h. HMGA proteins phosphorylation by DNA-PK was performed incubating 10 μg of recombinant protein with 100 Units of DNA-PK (Promega) in 50 μL of reaction volume (50 mM HEPES pH 7.5, 100 mM KCl, 1 mM MgCl_2_, 200 μM EGTA, 100 μM EDTA, 1 mM DTT, 1 μg/mL Calf Thymus DNA, 0.08 μg/μL BSA, and 3.3 μM [γ-^32^P] ATP or 200 μM ATP for LC-MS and LC-MS/MS analyses) at 30°C for 0.25, 0.5, 1, 2, or 16 h. For DNA-PK inhibition assay, a specific inhibitor for this enzyme was used (50 nM NU7441, Axon). Proteins phosphorylated in the presence of [γ-^32^P] ATP were separated by SDS-PAGE (T = 15%), the gel was Blue Coomassie stained, dried at 80°C, and exposed to autoradiography.

### LC/MS and LC-MS/MS analyses

These analyses were carried out essentially as previously described [28, 29].

### Double-stranded ligation assays

The double-stranded ligation assays were performed as previously described [33, 61, 62]. Briefly, a 442 bp ds DNA fragment with 4 bp overhangs at each end was produced from the Bluescript plasmid and was 5’ end-labeled with [γ-^32^P] ATP. The cohesive ends are not complementary to limit circularization. The indicated amounts of protein complexes were incubated with 20 ng of labeled DNA fragment for 30 min in 30 μl of reaction mixture (50 mM tri-ethanolamine, pH 7.5, 2 mM Mg(OAc)_2_, 2 mM dithiothreitol, 0.1 mg/ml bovine serum albumin, 12% polyethylene glycol). After incubation, the reactions were stopped by the addition of 1.5 μl of 10% SDS. Following deproteinization using the Qiaquick purification columns (Qiagen), the DNA was eluted in 30 μl of water and analyzed on a 0.8% agarose gel.

### Cell culture

The human breast cancer epithelial cell line MCF7 was grown in Dulbecco's MEM Nutrient Mix F12 (1:1) with 25 mM HEPES (DME/F12-HEPES, Euroclone) supplemented with 2 mM L-glutamine, 10% Tetracycline free fetal bovine serum (Tet-free FBS, Euroclone), penicillin (100 U/mL, Euroclone), streptomycin (100 μg/mL, Euroclone), and 1X MEM non essential amino acids (Sigma Aldrich). The human triple negative breast cancer cell line MDA–MB–231 was grown in Dulbecco's modified Eagle's medium (DMEM) containing 100 U/ml penicillin, 100 mg/ml streptomycin, 2 mM L–glutamine, and 10% tetracycline–free FBS. MDA-MB-231_shCTRL and MDA-MB-231_shA1_3 cells were described previously [11]. Cells were grown in humidified incubator at 37°C under 5% CO_2_ in air.

### MCF7 stable cell lines for HMGA1a overexpression

HMGA1a plasmid expression vector was obtained by subcloning the PCR products of the coding regions of human HMGA1a cDNA into the pcDNA3-HA plasmid expression vector (Invitrogen). pcDNA3-HA empty vector was chosen as a control. Plasmids were linearized at the *ScaI* restriction site. Vectors were dephosphorylated using Calf Intestinal Phosphatase and purified at each reaction step with Illustra^™^ GFX^™^ PCR DNA and Gel Band Purification kit (GE Healthcare). Plasmids were transfected into MCF7 cells using the Fugene reagent (Roche) following the manufacturer's instructions. 48 hours after transfection, confluent cells were detached by trypsinization and 8x10^5^ cells were seeded in 10 cm ø dishes. Clone selection was obtained by adding 1.5 mg/mL G418 disulfate salt (Sigma Aldrich) to the culture medium. Selection was carried out for 15 days. G418 concentration was gradually reduced at 200 μg/mL in culture maintenance phase. MCF7_CTRL and MCF7_HMGA1a pools were obtained by combining single clones.

### Western blot analyses of DNA damage induction in MCF7 stable cell lines

MCF7, MCF7_CTRL, and MCF7_HMGA1a were seeded at 2.5x10^5^ cells/3.5 cm ø plate. The day after seeding, DNA damage induction was conducted treating cells with 1 μM Doxorubicin (Sigma Aldrich) in DME/F12-HEPES for 2 hours. Doxorubicin containing medium was substituted with fresh one and DNA damage was monitored at different levels and time points, collecting total lysates (not treated, 2, 4, and 8 hours after treatment removal) with western blot analyses. Expression levels of H2AX and γ-H2AX were analyzed by western blot following standard procedures (α-actin b—Sigma Aldrich; α-H2AX—Millipore; α-γ-H2AX—Abcam).

### Immunofluorescence for γ-H2AX in MCF7 stable cell lines

DNA damage induction was conducted as described above. After treatment removal, cells were fixed with PFA 4% at different time points to detect DNA damage by foci formation. Presence of γ-H2AX foci was analyzed by immunofluorescence following standard procedures (α-γ-H2AX—Abcam; Alexa Fluor 488 green anti-mouse—Invitrogen).

### Colony formation assay

MDA-MB-231_shCTRL, MDA-MB-231_shA1_3, MCF7_CTRL, and MCF7_HMGA1a cells were incubated with doxorubicin at different concentrations for 2 hours, to induce DNA damage (not treated (NT), 0.5 μM and 1 μM–MCF7 cells, 10 nM and 100 nM–MDA-MB-231 cells). Two (MCF7) or one (MDA-MB-231) hour later cells were detached, counted, and reseeded in standard medium in quadruplicate for each condition and cellular type (MCF7: 5x10^3^ cells/6 cm ø dishes; MDA-MB-231: 600 cells/6 cm ø dishes). Cells returned to incubator for 10 (MCF7) or 14 (MDA-MB-231) days to form colonies and then were fixed with cold methanol for 20 minutes, dried, and stained with 0.5% crystal violet in 25% methanol for 20 minutes. After washing with water and drying, colonies were counted manually.

### Comet assay

MDA-MB-231_shCTRL and MDA-MB-231_shA1_3 cells were treated with 10nM doxorobucin. MCF7_CTRL and MCF7_HMGA1a cells were treated with 1 μM doxorubicin. All cell lines tested were treated for 2 hours, then collected 3 and 5 hours after treatment removal, and processed for comet assay. A Neutral Comet Assay was performed according to the manufacturer's protocol (Trevigen kit; 4250-050-K). Cells were washed in ice cold PBS, scraped, combined with molten LM agarose, and immediately pipetted onto CometSlide. Slides were treated with Lysis Solution overnight and then in Neutral Electrophoresis Buffer. After electrophoresis, slides were treated with DNA Precipitation Solution and fixed in 70% ethanol, dried, and stained with SYBR green. Comets were visualized by fluorescence microscopy and analyzed using ImageJ open source software using the OpenComet plugin.

### Statistics

Statistical significance was evaluated using a 2-tailed Student's *t* test. P<0.05 was considered significant. All bar graph data shown represent mean ± s.d.

## Supporting Information

S1 Fig Evaluation of HMGA1a expression levels and cell cycle phase distribution in MCF7_CTRL and MCF7_HMGA1a cells. A. Western blot analysis showing the expression level of endogenous HMGA1a/b proteins (lower band) and HA-tagged HMGA1a (upper band) in MCF7_CTRL and MCF7_HMGA1a (α-HMGA1 antibody). Both not treated (NT) cells and cells treated with 1 μM doxorubicin for 2 hours and left to recover for 0, 1, 2, 4, and 6 hours are shown. Protein loading normalization is assessed by checking actin level (α-actin antibody). Protein molecular markers are shown on the left. B. Cell cycle phase distribution (%) of MCF7, MCF7_CTRL, and MCF7_HMGA1a cells obtained by FACS analysis. C. Western blot analysis showing the expression level of endogenous HMGA1a/b proteins on MDA-MB-231_shCTRL and MDA-MB-231_shA1_3. Protein loading normalization is assessed by checking actin level (α-actin antibody). Protein molecular markers are shown on the left.
(PDF)

S2 Fig HMGA1a modulates DNA-PK autophosphorylation. DNA-PK was activated for phosphorylation in the presence (lanes 2, 4, 6, 8, and 10) or absence (lanes 1, 3, 5, 7, and 9) of HMGA1a protein. Phosphorylation reactions were made in the presence of [γ-^32^P] ATP for 5 min, 15 min, 30 min, 60 min and 16 h. Phosphorylated proteins were separated by SDS-PAGE (T = 7.5 and 15% for the separation of high and low molecular weight phosphorylated substrates, respectively) and ^32^P incorporation visualized by autoradiography. Protein molecular markers (kDa) are indicated on the left.
(PDF)

S1 MS Data File LC–MS/MS analyses data. (PDF)
