# Supplementary figures and images for: The Architectural Chromatin Factor High Mobility Group A1 Enhances DNA Ligase IV Activity Influencing DNA Repair
Source: PLoS One. 2016 Oct 10;11(10):e0164258. doi: 10.1371/journal.pone.0164258 (PMC5056749; doi:10.1371/journal.pone.0164258)

# Supporting Figure 1

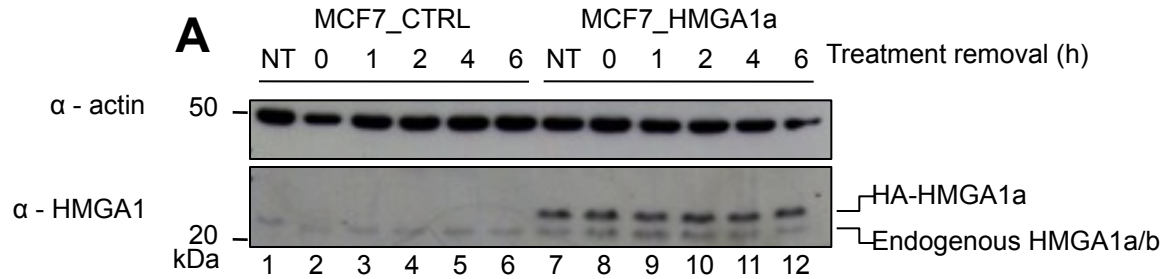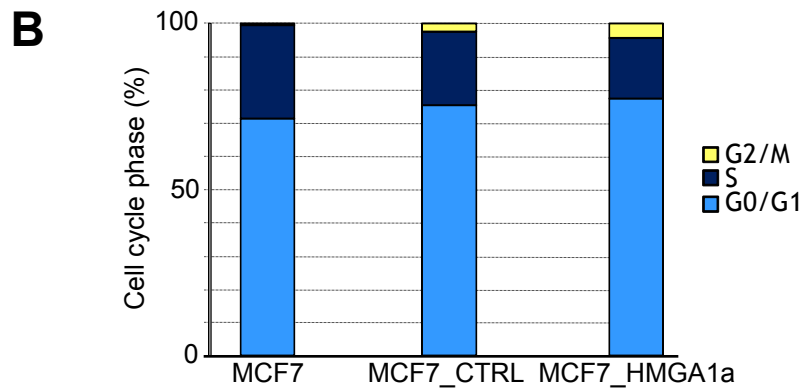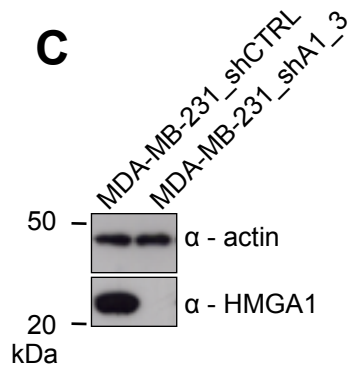

Supplement: S1 Fig — A. Western blot analysis showing the expression level of endogenous HMGA1a/b proteins (lower band) and HA-tagged HMGA1a (upper band) in MCF7_CTRL and MCF7_HMGA1a (α-HMGA1 antibody). Both not treated (NT) cells and cells treated with 1 μM doxorubicin for 2 hours and left to recover for 0, 1, 2, 4, and 6 hours are shown. Protein loading normalization is assessed by checking actin level (α-actin antibody). Protein molecular markers are shown on the left. B. Cell cycle phase distribution (%) of MCF7, MCF7_CTRL, and MCF7_HMGA1a cells obtained by FACS analysis. C. Western blot analysis showing the expression level of endogenous HMGA1a/b proteins on MDA-MB-231_shCTRL and MDA-MB-231_shA1_3. Protein loading normalization is assessed by checking actin level (α-actin antibody). Protein molecular markers are shown on the left. (PDF) [file pone.0164258.s001.pdf]

## Supporting Figure 2

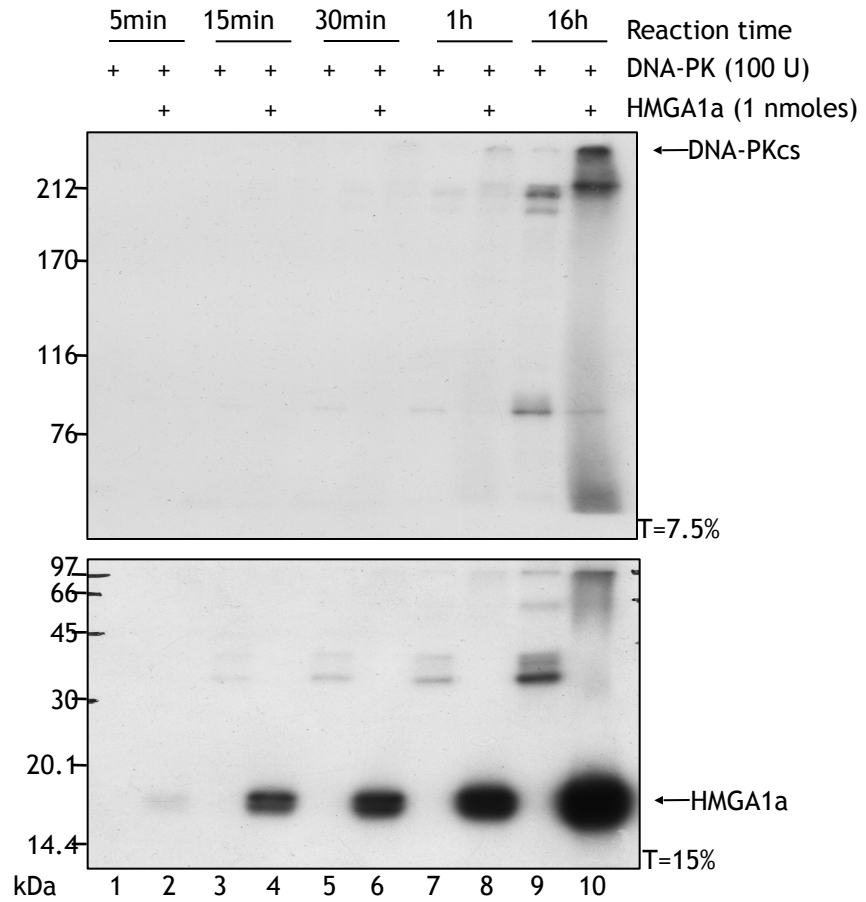

Supplement: S2 Fig — DNA-PK was activated for phosphorylation in the presence (lanes 2, 4, 6, 8, and 10) or absence (lanes 1, 3, 5, 7, and 9) of HMGA1a protein. Phosphorylation reactions were made in the presence of [γ-32P] ATP for 5 min, 15 min, 30 min, 60 min and 16 h. Phosphorylated proteins were separated by SDS-PAGE (T = 7.5 and 15% for the separation of high and low molecular weight phosphorylated substrates, respectively) and 32P incorporation visualized by autoradiography. Protein molecular markers (kDa) are indicated on the left. (PDF) [file pone.0164258.s002.pdf]
