# Supplementary material for: The Architectural Chromatin Factor High Mobility Group A1 Enhances DNA Ligase IV Activity Influencing DNA Repair
Source: PLoS One. 2016 Oct 10;11(10):e0164258. doi: 10.1371/journal.pone.0164258 (PMC5056749; doi:10.1371/journal.pone.0164258)
Supplement: S1 MS Data File — (PDF) [file pone.0164258.s003.pdf]

Supplementary MS Data: HMGA1a FL – LC–MS/MS analysis data (summary)

a) HMGA1a aminoacidic sequence (S/T residues evidenced in red, SQ consensus sites evidenced in blue)

SESSSKSSQP LASKQEKDGT EKRGRGRPRK QPPVSPGTAL VGSQKEPSEV PTPKRPRGRP KGSKNKGAAK TRKTTTTPGR KPRGRPKKLE KEEEEGISQE SSEEEQ

---

b) Peptides detected in LC–MS/MS analysis (HMGA1a full length (FL) tryptic digestion – Mascot search results). The phosphorylation sites assigned by Mascot are evidenced In green.

Observed	Mr(expt)	Mr(calc)	Score	Expect	Peptide (aa start–stop position)
367.13	732.2454	732.3766	16	0.43	K.TTTTPGR.K (74–80)
409.16	816.3054	816.4341	57	0.00046	K.SSQPLASK.Q (7–14)
418.15	834.2854	834.2830	11	0.82	MSESSSK.S + P (Nter-6)
861.25	860.2427	860.4716	3	1.1	R.KTTTTPGR.K (73–80)
431.15	860.2854	860.4716	18	1.2	R.KTTTTPGR.K (73–80)
449.15	896.2854	896.4004	31	0.19	K.SSQPLASK.Q + P (7–14)
492.22	982.4254	982.4971	40	0.01	K.EPSEVPTPK.R (46–54)
532.22	1062.4254	1062.4634	37	0.031	K.EPSEVPTPK.R + P (46–54)
558.58	1672.7182	1672.8549	6	2.3	R.KQPPVSPGTALVGSQK.E + P (30–45)
733.33	1464.6454	1464.7937	50	0.00014	K.QPPVSPGTALVGSQK.E (31–45)
489.23	1464.6682	1464.7937	21	0.57	K.QPPVSPGTALVGSQK.E (31–45)
773.31	1544.6054	1544.7600	57	2.6e-05	K.QPPVSPGTALVGSQK.E + P (31–45)
531.89	1592.6482	1592.8886	48	0.0021	R.KQPPVSPGTALVGSQK.E (30–45)
837.35	1672.6854	1672.8549	51	0.00016	R.KQPPVSPGTALVGSQK.E + P (30–45)
869.74	1737.4654	1737.6700	97	4.8e-08	K.EEEEGISQESSEEEQ.- (92–Cter)
580.18	1737.5182	1737.6700	43	0.0047	K.EEEEGISQESSEEEQ.- (92–Cter)
906.66	2716.9582	2717.3078	16	0.26	R.KQPPVSPGTALVGSQKEPSEVPTPK.R + 2P (30–54)
703.57	2107.6882	2107.8916	55	0.00028	K.LEKEEEEGISQESSEEEQ.- (89–106)
746.27	2235.7882	2235.9866	71	2.2e-05	K.KLEKEEEEGISQESSEEEQ.- (88–106)
837.34	2508.9982	2509.2465	52	5.2e-05	K.QPPVSPGTALVGSQKEPSEVPTPK.R + P (31–54)
880.03	2637.0682	2637.3415	50	0.0002	R.KQPPVSPGTALVGSQKEPSEVPTPK.R + P (30–54)

---

c) Peptide intensities (cps) extracted by LC–MS/MS analysis by generating extracted ion chromatograms (EIC) for each indicated m/z value. Most probable phosphorylated site (Mascot site analysis) evidenced in green. HMGA1a FL analysis.

m/z	z	peptide	Phosphate	Sequence	Intensity (cps)	Relative intensity
409.16	2	7–14	0	SSQPLASK	413192960	100
449.15	2		1		136824048	33
492.22	2	46–54	0	EPSEVPTPK	1148811776	100
532.22	2		1		55826432	4.9
733.33	2	31–45	0	QPPVSPGTALVGSQK	551830464	100
773.31	2		1		32568356	5.9
797.35	2	30–45	0	KQPPVSPGTALVGSQK	150227632	100
837.35	2		1		142235120	95
869.74	2	92–106	0	EEEEGISQESSEEEQ	126906968	100
909.74	2		1		15437390	12.2
853.36	3	30–54	0	KQPPVSPGTALVGSQKEPSEVPTPK	nd	0
880.03	3		1		264766768	100
810.67	3	31–54	0	QPPVSPGTALVGSQKEPSEVPTPK	nd	0
837.34	3		1		83190216	100

---

d) Example of phosphorylation site assignment by Mascot analysis

Peptide K.QPPVSPGTALVGSQK.E + P (31–45), Mr(calc): 1544.76, Variable modifications: pS13 (number refers to the position within the peptide), Matches : 63/240 fragment ions using 78 most intense peaks.

Score	Mr(calc)	Delta	Sequence	Site analysis
57.2	1544.76	–0.1545	QPPVSPGTALVG**pS**QK	S13 99.82%
29.2			QPPVSPG**pT**ALVGSQK	T8 0.16%
20.9			QPPV**pS**PGTALVGSQK	S5 0.02%

Supplementary MS Data: HMGA2 FL – LC–MS/MS analysis data (summary)

a) HMGA2 aminoacidic sequence (S/T residues evidenced in red, SQ consensus sites evidenced in blue)

SARGEGAGQP STSAQGQPAA PAPQKRGRGR PRKQQQEPTG EPSPKRPRGR PKGSKNKSPS KAAQKKAEAT GEKRPRGRPR KWPQQVVQKK PAQEETEETS SQESAEED

---

b) Peptides detected in LC–MS/MS analysis (HMGA2 full length (FL) tryptic digestion – Mascot search results). The phosphorylation sites assigned by Mascot are evidenced In green.

Observed	Mr(expt)	Mr(calc)	Score	Expected	Peptide (aa start–stop position)
417.20	832.3854	832.4290	46	0.0052	K.KAEATGEK.R (66–73)
506.72	1011.4254	1011.5502	33	0.089	K.WPQQVVQK.K (82–89)
380.83	1139.4682	1139.6451	36	0.09	R.KWPQQVVQK.K (81–89)
570.81	1139.6054	1139.6451	30	0.07	R.KWPQQVVQK.K (81–89)
663.32	1324.6254	1324.6259	23	0.036	K.QQQEPTGEPSPK.R (34–45)
485.20	1452.5782	1452.7209	30	0.24	R.KQQQEPTGEPSPK.R (33–45)
727.30	1452.5854	1452.7209	63	3.7e-05	R.KQQQEPTGEPSPK.R (33–45)
735.60	2203.7782	2202.8325	28	0.011	K.KPAQEETEETSSQESAEED.- + P (90–Cter)
767.34	1532.6654	1532.6872	33	0.028	R.KQQQEPTGEPSPK.R + P (33–45)
678.94	2033.7982	2033.9766	56	0.00031	R.GEGAGQPSTSAQGQPAAPAPQK.R (4–25)
1017.94	2033.8654	2033.9766	92	2.5e-08	R.GEGAGQPSTSAQGQPAAPAPQK.R (4–25)
708.60	2122.7782	2122.8662	73	5.5e-07	K.KPAQEETEETSSQESAEED.- (90–Cter)
735.21	2202.6082	2202.8325	27	0.011	K.KPAQEETEETSSQESAEED.- + P (90–Cter)
1102.36	2202.7054	2202.8325	78	2.4e-07	K.KPAQEETEETSSQESAEED.- + P (90–Cter)
735.25	2202.7282	2202.8325	45	0.0024	K.KPAQEETEETSSQESAEED.- + P (90–Cter)

---

c) Peptide intensities (cps) extracted by LC–MS/MS analysis by generating extracted ion chromatograms (EIC) for each indicated m/z value. Most probable phosphorylated site (Mascot site analysis) evidenced in green. HMGA2 FL analysis.

m/z	z	peptide	Phosphate	Sequence	Intensity (cps)	Relative intensity
727.30	2	33–45	0	KQQQEPTGEPSPK	61564724	100
767.34	2		1		9513640	15.45
485.20	3	33–45	0	KQQQEPTGEPSPK	153117008	100
511.88	3		1		7362616	4.8
708.60	3	90–108	0	KPAQEETEETSSQESAEED	31226180	32.8
735.21	3		1		95052592	100

---

d) Examples of phosphorylation site assignment by Mascot analysis

Peptide R.KQQQEPTGEPSPK.R + P (33–45), Mr(calc): 1532.68, Variable modifications: pS11 (number refers to the position within the peptide), Matches : 50/204 fragment ions using 83 most intense peaks.

Score	Mr(calc)	Delta	Sequence	Site analysis
33.3	1532.68	–0.0217	KQQQEPTGEP**pS**PK	S11 99.42%
10.9	1532.68	–0.0217	KQQQEP**pT**GEPSPK	T8 0.16%

Peptide K.KPAQEETEETSSQESAEED.- + P (90–Cter), Mr(calc): 2202.8325, Variable modifications: pS12 (number refers to the position within the peptide), Matches : 49/304 fragment ions using 65 most intense peaks.

Score	Mr(calc)	Delta	Sequence	Site analysis
78.0	2202.83	–0.01270	KPAQEETEETS**pS**QESAEED	S12 90.10
66.5			KPAQEETEET**pS**SQESAEED	S11 6.32%
62.2			KPAQEETEE**pT**SSQESAEED	T10 2.38%
59.2			KPAQEETEETSSQE**pS**AEED	S15 1.18%
41.2			KPAQEE**pT**EETSSQESAEED	T7 0.02%

Peptide K.KPAQEETEETSSQESAEED.- + P (90–Cter), Mr(calc): 2202.8325, Variable modifications: pS15 (number refers to the position within the peptide), Matches : 49/300 fragment ions using 80 most intense peaks.

Score	Mr(calc)	Delta	Sequence	Site analysis
26.9	2202.83	–0.01270	KPAQEETEETS**SQE**p**S**AEED	S15 44.02
26.4			KPAQEETEETS**pS**QESAEED	S12 38.87
21.2			KPAQEETEET**pS**SQESAEED	S11 11.77
17.6			KPAQEETEE**pT**SSQESAEED	T10 5.14

DNA–PK tryptic digestion and LC–MS/MS analysis – Mascot Search Results

Observed	Mr(expt)	Mr(calc)	Delta	Score	Expect	Peptide
**1) ROA1_HUMAN**	**279**	**Heterogeneous nuclear ribonucleoprotein A1**				
525.2500	1048.4854	1048.4502	0.0353	45	0.0044	R.DYFEQYGK.I
609.8100	1217.6054	1217.6326	-0.0271	80	3.3e-06	K.IEVIEIMTDR.G
719.3900	1436.7654	1436.7372	0.0283	22	0.1	R.EDSQRPGAHLTVK.K
847.5200	1693.0254	1693.6928	-0.6674	104	3.8e-09	R.NQGGYGGSSSSSSYGSGR.R
850.4200	1698.8254	1698.7526	0.0728	68	6.6e-06	R.GFAFVTFDDHDSVDK.I
892.9600	1783.9054	1783.8992	0.0062	115	3.6e-10	K.LFIGGLSFETTDESLR.S
**2) PRKDC_HUMAN**	**203**	**DNA-dependent protein kinase catalytic subunit**				
446.2600	890.5054	891.4450	-0.9396	14	0.39	K.AAYLSDPR.A
492.7500	983.4854	983.4437	0.0417	112	3.2	R.ASGEVRTGK.W + P (ST)
498.3100	994.6054	994.6426	-0.0372	39	0.008	K.IPALDLLIK.L
506.8000	1011.5854	1011.5865	-0.0011	24	0.19	K.FVPLLPGNR.S
568.3400	1134.6654	1134.6033	0.0621	46	0.0027	R.HGDLPDIQIK.H
582.3600	1162.7054	1162.6185	0.0869	12	5.1	R.ICSKPVVLPK.G + P (ST)
647.3200	1292.6254	1292.6725	-0.0470	42	0.0041	R.DQNILLGTTYR.I
698.9200	1395.8254	1395.6882	0.1373	55	0.00017	K.LNESTFDTQITK.K
722.9400	1443.8654	1443.8337	0.0318	97	6.6e-08	K.NLLIFENLIDLK.R
752.4100	1502.8054	1502.6525	0.1529	9	3	K.QDWVDGEPTEAEK.D
848.5000	1694.9854	1694.9131	0.0723	87	3e-07	R.EFFSTIVVDAIDVLK.S
737.7200	2210.1382	2210.1136	0.0245	9	2	R.EFLKWSIKQITPQQQEK.S + P (ST)
752.7900	2255.3482	2254.9313	0.4168	6	3.1	K.SRFTKLNESTFDTQITK.K + 3 (ST)
**3) GCN1L_HUMAN**	**119**	**Translational activator GCN1**				
638.1200	1911.3382	1911.0201	0.3181	28	0.026	K.VLPLEALVTDAGEVTEAGK.A
802.5800	1603.1454	1603.9548	-0.8094	39	0.005	K.ILPEIIPILEEGLR.S
707.1200	2118.3382	2117.1732	1.1650	47	0.00023	R.NPEILAIAPVLLDALTDPSR.K
746.4400	2236.2982	2236.2566	0.0416	89	3.2e-07	R.LQELDGELEAALGLLDIILAK.N
**4) ALBU_HUMAN**	**112**	**Serum albumin**				
464.2900	926.5654	926.4861	0.0793	31	0.061	K.YLYEIAR.R
609.8100	1217.6054	1217.5264	0.0791	14	15	K.CASLQKFGER.A + P (ST)
756.4200	1510.8254	1510.8355	-0.0101	37	0.0042	K.VPQVSTPTLVEVSR.N
547.3300	1638.9682	1638.9305	0.0377	53	0.00018	K.KVPQVSTPTLVEVSR.N
820.5100	1639.0054	1638.9305	0.0750	75	5.9e-07	K.KVPQVSTPTLVEVSR.N
**5) H10_HUMAN**	**86**	**Histone H1.0**				
530.2400	1058.4654	1059.5196	-1.0542	19	0.24	K.VGENADSQIK.L
651.3000	1300.5854	1300.6623	-0.0768	72	4.9e-06	M.TENSTSAPAAKPK.R
**6) HNRPD_HUMAN**	**85**	**Heterogeneous nuclear ribonucleoprotein D0**				
744.9000	1487.7854	1487.7508	0.0347	85	2.9e-07	K.IFVGGLSPDTPEEK.I
**7) E2AK2_HUMAN**	**82**	**Interferon-induced, double-stranded RNA-activated protein kinase**				
702.4400	1402.8654	1402.8071	0.0583	82	3.4e-07	K.VLALELFEQITK.G
**8) NONO_HUMAN**	**67**	**Non-POU domain-containing octamer-binding protein**				
543.2400	1084.4654	1085.5539	-1.0885	49	0.00044	K.VELDNMPLR.G
890.1100	2667.3082	2667.3180	-0.0099	50	0.0024	R.NLPQYVSNELLEEAFSVFGQVER.A
**9) DDX17_HUMAN**	**66**	**Probable ATP-dependent RNA helicase DDX17**				
613.4100	1224.8054	1225.7030	-0.8976	66	7.3e-05	K.APILIATDVASR.G
**10) EF1A1_HUMAN**	**57**	**Elongation factor 1-alpha 1**				
488.3200	974.6254	974.5437	0.0818	57	0.0006	R.LPLQDVYK.I
733.7200	2198.1382	2199.0517	-0.9136	10	1.2	K.MDSTEPPYSQKRYEEIVK.E
**11) XRCC6_HUMAN**	**47**	**X-ray repair cross-complementing protein 6**				
503.3400	1004.6654	1004.5542	0.1112	47	0.0051	R.ILELDQFK.G
677.8800	1353.7454	1353.7180	0.0274	15	0.26	R.DLLAVVFYGTEK.D
782.0300	2343.0682	2343.1634	-0.0953	19	0.77	R.NIPPYFVALVPQEEELDDQK.I
**12) HNRPQ_HUMAN**	**45**	**Heterogeneous nuclear ribonucleoprotein Q**				
587.3300	586.3227	587.3027	-0.9800	1	0.81	R.GGNVGGK.R
797.4200	1592.8254	1592.7974	0.0281	45	0.0022	R.DLFEDELVPLFEK.A
**13) CAND1_HUMAN**	**43**	**Cullin-associated NEDD8-dissociated protein 1**				
522.8600	1043.7054	1042.6209	1.0846	4	1.8	K.CLGPLVSKVK.E
503.8700	1508.5882	1509.6663	-1.0781	43	0.0046	R.ATCTTKVKANSVK.Q + 2 P (ST)
822.3300	1642.6454	1642.7426	-0.0971	8	1.1	-.MASASYHISNLLEK.M + P (ST)
**14) DNJC2_HUMAN**	**39**	**DnaJ homolog subfamily C member 2**				
823.9200	1645.8254	1645.7777	0.0478	39	0.00047	K.DNFFEVFTPVFER.N
**15) HNRPL_HUMAN**	**37**	**Heterogeneous nuclear ribonucleoprotein L**				
454.2500	906.4854	906.4494	0.0360	32	0.023	R.MGPPVGGHR.R
501.7500	1001.4854	1001.4203	0.0652	28	0.008	R.YYGGGSEGGR.A
**16) MSH2_HUMAN**	**33**	**DNA mismatch repair protein Msh2**				
785.9900	1569.9654	1569.9494	0.0160	33	0.0062	K.LLLAVFVTPLTDLR.S
788.3000	1574.5854	1574.8780	-0.2926	6	2.4	K.DIYQDLNRLLKGK.K
**17) HMGA2_HUMAN**	**31**	**High mobility group protein HMGI-C**				
570.8600	1139.7054	1139.6451	0.0603	31	0.03	R.KWPQQVVQK.K
727.3400	1452.6654	1452.7209	-0.0554	25	0.12	R.KQQQEPTGEPSPK.R
**18) CAPR1_HUMAN**	**26**	**Caprin-1**				
660.3800	1318.7454	1319.7337	-0.9882	26	0.052	K.TVLELQYVLDK.L
**19) HEMGN_HUMAN**	**23**	**Hemogen**				
505.3000	1008.5854	1008.4529	0.1326	23	0.047	K.TEPPGSITK.V + Phospho (ST)
**20) SFPQ_HUMAN**	**20**	**Splicing factor, proline- and glutamine-rich**				
625.8900	1874.6482	1874.9992	-0.3510	10	0.91	R.QLRVRFATHAAALSVR.N + P (ST)
671.8000	1341.5854	1340.6586	0.9269	20	0.047	R.FGQGGAGPVGGQGPR.G
746.6600	2236.9582	2235.8816	1.0766	7	0.88	R.FAQHGTFEYEYSQRWK.S + 2 P (ST)

Supplementary References regarding the proteins identified with DNA–PK tryptic digestion and LC–MS/MS analysis that have a link with DNA–PK.

Heterogeneous nuclear ribonucleoprotein A1:

1: Ting NS, Pohorelic B, Yu Y, Lees-Miller SP, Beattie TL. The human telomerase RNA component, hTR, activates the DNA-dependent protein kinase to phosphorylate heterogeneous nuclear ribonucleoprotein A1. Nucleic Acids Res. 2009 Oct;37(18):6105-15. doi: 10.1093/nar/gkp636. Epub 2009 Aug 5. PubMed PMID:19656952; PubMed Central PMCID: PMC2764450.

2: Sui J, Lin YF, Xu K, Lee KJ, Wang D, Chen BP. DNA-PKcs phosphorylates hnRNP-A1 to facilitate the RPA-to-POT1 switch and telomere capping after replication. Nucleic Acids Res. 2015 Jul 13;43(12):5971-83. doi: 10.1093/nar/gkv539. Epub 2015 May 20. PubMed PMID: 25999341.

Histone H1.0

1: Rosidi B, Wang M, Wu W, Sharma A, Wang H, Iliakis G. Histone H1 functions as a stimulatory factor in backup pathways of NHEJ. Nucleic Acids Res. 2008 Mar;36(5):1610-23. doi: 10.1093/nar/gkn013. Epub 2008 Feb 3. PubMed PMID: 18250087; PubMed Central PMCID: PMC2275134.

2: Kysela B, Chovanec M, Jeggo PA. Phosphorylation of linker histones by DNA-dependent protein kinase is required for DNA ligase IV-dependent ligation in the presence of histone H1. Proc Natl Acad Sci U S A. 2005 Feb 8;102(6):1877-82. Epub 2005 Jan 25. PubMed PMID: 15671175; PubMed Central PMCID: PMC548527.

Non-POU domain-containing octamer-binding protein and Splicing factor, proline- and glutamine-rich

1) Bladen CL, Udayakumar D, Takeda Y, Dynan WS. Identification of the polypyrimidine tract binding protein-associated splicing factor.p54(nrb) complex as a candidate DNA double-strand break rejoining factor. J Biol Chem. 2005 Feb 18;280(7):5205-10. Epub 2004 Dec 7. PubMed PMID: 15590677.

2) Udayakumar D, Dynan WS. Characterization of DNA binding and pairing activities associated with the native SFPQ·NONO DNA repair protein complex. Biochem Biophys Res Commun. 2015 Aug 7;463(4):473-8. doi: 10.1016/j.bbrc.2015.05.024. Epub 2015 May 18. PubMed PMID: 25998385; PubMed Central PMCID: PMC4497844.
